# Emotional States Modulate the Recognition Potential during Word Processing

**DOI:** 10.1371/journal.pone.0047083

**Published:** 2012-10-08

**Authors:** Taomei Guo, Min Chen, Danling Peng

**Affiliations:** State Key Laboratory of Cognitive Neuroscience and Learning, Beijing Normal University, Beijing, People's Republic of China; National University of Singapore, Singapore

## Abstract

This study examined emotional modulation of word processing, showing that the recognition potential (RP), an ERP index of word recognition, could be modulated by different emotional states. In the experiment, participants were instructed to compete with pseudo-competitors, and via manipulation of the outcome of this competition, they were situated in neutral, highly positive, slightly positive, highly negative or slightly negative emotional states. They were subsequently asked to judge whether the referent of a word following a series of meaningless character segmentations was an animal or not. The emotional induction task and the word recognition task were alternated. Results showed that 1) compared with the neutral emotion condition, the peak latency of the RP under different emotional states was earlier and its mean amplitude was smaller, 2) there was no significant difference between RPs elicited under positive and negative emotional states in either the mean amplitude or latency, and 3) the RP was not affected by different degrees of positive emotional states. However, compared to slightly negative emotional states, the mean amplitude of the RP was smaller and its latency was shorter in highly negative emotional states over the left hemisphere but not over the right hemisphere. The results suggest that emotional states influence word processing.

## Introduction

Emotion plays an important role in our everyday lives and interacts with our cognition [1]. Two lines of empirical evidence suggest that emotion may influence language processing. A number of previous studies have examined the emotional modulation of language processing by comparing the differences between processing words with various emotional valences. Others have examined the issue by using emotional stimuli such as pictures or music to induce different types of emotional states in participants. These studies are reviewed below, with a particular focus on those using event related potentials (ERPs), as in the present study.

Emotional words contain both conceptual and emotional meanings, making these words particularly useful for research on emotional modulation of language processing. Previous studies using behavioral measures have shown that participants more accurately recognize negative words compared to positive and neutral words in a word recognition task, indicating a facilitation effect of negative emotional information on word processing [2]. Recent ERP studies have also shed light on the influence of emotional information on different stages of word processing. Emotional words with both positive and negative valences were found to elicit a larger early posterior negativity (EPN) than neutral words approximately 250 ms after stimulus onset [3]–[7]. Furthermore, previous studies have shown that positive or negative words, or both, elicited a greater late positive complex (LPC) than neutral words approximately 500 ms after stimulus onset [3]–[9]. An enhanced EPN may reflect a benefit from early automatic allocation of attention to the processing of emotional words, while an enhanced LPC may indicate more the allocation of more attentional resources for conscious level processing of words influenced by emotional information [6].

While these studies imply an effect of emotional words on word processing itself, other studies have also found that emotional states or backgrounds influence language processing at large. These studies first induced emotional states in participants with non-linguistic methods and subsequently employed a linguistic task to examine the effects of emotion on processing. For instance, Olafson and Richard (2001) [10] used musical segments to induce emotional states and then examined their effects on word judgment. Their results showed that participants were faster to respond to negative words under a sad emotional state and positive words under a happy emotional state. ERP studies have also found an influence of emotional states or backgrounds on language processing. For example, Federmeier et al. (2001) [11] first induced positive or neutral emotional states by presenting different types of emotionally arousing pictures, followed by a sentence-reading task. Federmeier and colleagues found that the mean amplitude of the N400 was positively correlated with the extent of incongruence between final words and sentences under a neutral emotional state, while this correlation was attenuated under a positive emotional state. The authors proposed a facilitation effect of positive emotional states on the processing of unexpected words. In a more recent study by Ihssen et al. (2007) [12], words were alternately presented with either “high” or “low” emotionally arousing pictures, but participants were only required to perform a lexical decision task. Results showed that a smaller N1 ERP component with delayed peak latency and a smaller LPC were elicited when words were presented after high arousing pictures compared to neutral pictures. The difference between low and neutral arousing pictures was not significant. These findings revealed an interference effect of highly arousing emotional backgrounds on word processing. The authors proposed that the emotional background may have captured more attentional resources, resulting in a lack of such resources for subsequent word processing.

According to the literature, evidence for emotional modulation on language processing has been found in studies employing both linguistic and non-linguistic emotional stimuli. ERP studies with emotional words have consistently found a facilitation effect of emotional words, especially negative words, compared to neutral words [3], [5], [9]. However, mixed findings were shown in studies on language processing under different emotional backgrounds. Specifically, some studies found a facilitation effect of emotional backgrounds [11], while others found an interference effect [12]. Different results may arise from the different methods used to induce emotional states. When emotional stimuli such as pictures are presented in a separate session to induce emotion, they are not likely to interfere with subsequent word processing. However, when emotional stimuli are presented in an alternating fashion with words, they appear to capture more attention and cause interference in word processing. Furthermore, early emotional modulation of word processing has been revealed by different ERP components such as N1 and EPN. However, it is not clear whether these ERP effects are specific to word processing per se or early visual perception of visually presented stimuli. It is therefore worth examining whether semantic processing of word recognition is modulated by emotion.

In addition to the well-known N400 ERP component related to semantic integration [13]–[14], another ERP component sensitive to language processing is the recognition potential (RP). The RP is an electrical brain response with a peak around 200 to 250ms, which is typically elicited by recognizable visual stimuli such as words, pictures or faces but not by meaningless images or unfamiliar languages [15]–[17]. Previous studies have shown that the RP is sensitive to orthographic processing. For example, by comparing RPs evoked by semantically correct words, orthographically correct words and random letter strings, Martín-Loeches et al. (1999) [18] found that the RP elicited by the semantically correct words was strongest while that by the random letter strings was weakest. This observation suggested that the more similar a stimulus is to a real word, the stronger the evoked RP. These results were replicated by Rudell and Hu (2000) [19], which used high and low frequency letter strings with differing degrees of orthographic regularity as experimental stimuli. They found that high frequency letter strings evoked a larger RP even in tasks which did not require word processing. More recent studies have revealed that the RP may reflect semantic processing to some extent. For example, in the study of Martín-Loeches et al. (2001a) [20], animal words were found to evoke a larger RP compared to non-animal words. A similar study [21] found that concrete words evoked a larger RP than abstract words. Martín-Loeches (2007) [22] claimed that RP is sensitive to semantic aspects of the stimuli as semantic information may initiate the perceptual stage of word form analysis. Taken together, these studies suggest that the RP is a reliable index of word recognition. However, the studies reviewed so far have only investigated the cognitive mechanisms of the RP. An interesting question is whether the RP, as a sensitive index of word recognition, should also be influenced by emotional states. If so, how do different emotional states modulate the RP? An investigation of these questions will contribute to our understanding of the emotional modulation of language processing. The present study is thus aimed at examining these questions.

To induce different emotional states, a method commonly used in previous studies is the presentation of emotional pictures [11]–[12] or musical segments [10]. This method is useful for emotional induction, but it is stimulus driven. The present study attempted to use a task driven method to induce emotional states. Specifically, participants were informed that they would compete with another person (i.e., a competitor), and were further informed that they would receive payment only in the case that they achieved better results than their competitor. However, this “competitor” did not actually exist; results for the pseudo-competitor were generated prior to the experimental session, and all participants were shown the same set of pseudo-competitor results. Thus, participants' emotions were induced through a pseudo-competition task. Although this task involved more complex processing than simply reviewing emotional pictures, previous studies have demonstrated that emotional states can be triggered by tasks in which participants' choices are linked to monetary gains or losses [23]–[24]. The emotional induction task was alternated with a word recognition task, which was used to elicit the RP. A likely benefit of this alternation is stability of the induced emotional state, as the word recognition task immediately follows the induction task and lasts approximately 5 seconds. To prevent participants from becoming aware of the purpose of the emotion induction task, we did not ask participants to rate their mood during the experiment. Instead, they were required to do this at the end of the entire experiment. By using this method, the present study aims to examine whether and how the emotional states modulate the RP.

## Methods

### 2.1. Ethics Statement

This study was approved by the Institutional Review Board of the Imaging Center for Brain Research of Beijing Normal University. All participants gave informed, written consent prior to the experiment, and were paid for their participation.

### 2.2. Participants

Twenty-five native Chinese speakers (12 females) aged from 17 to 25 years participated in the experiment. All participants were right-handed, with normal or corrected-to-normal vision. No one reported neurological disorders. The data of three participants were excluded because of artifacts. The data of an additional 3 participants were excluded because they failed to monitor the results of their pseudo-competitor in the emotion induction task. The data of 19 participants were included for final analyses.

### 2.3. Stimuli

There were three types of stimuli in the word recognition task: non-animal words, animal words, and word segmentations. All stimuli consisted of two Chinese characters. The non-animal words were the test stimuli whose ERPs were of interest, but to which participants were not required to respond. There were in total 66 non-animal words. The animal words (target stimuli) were used to provide subjects with an active word processing task (i.e., to maintain their attention). As such, accuracy rates for these words were interpreted as a measure of attention. We were not interested in brain responses to the animal words. There were 42 animal words in total, and participants were required to press a button when an animal word was presented. The background stimuli were composed of the fragments of another 132 non-animal words, which were matched in visual attributes with Chinese characters.

### 2.4. Procedure

All stimuli were randomly presented in the center of a 19-inch screen with 24-point font size. Participants were seated in a quiet room at a distance of approximately 80 cm from the computer screen. Characters were 1.43° high by 1.43° wide. Participants were instructed to avoid eye blinks and body movements during the experiment. The order of stimulus presentation was programmed and controlled by the E-Prime Software (Psychology Software Tools). The refresh rate of the computer screen was 60 Hz.

**Figure 1:**
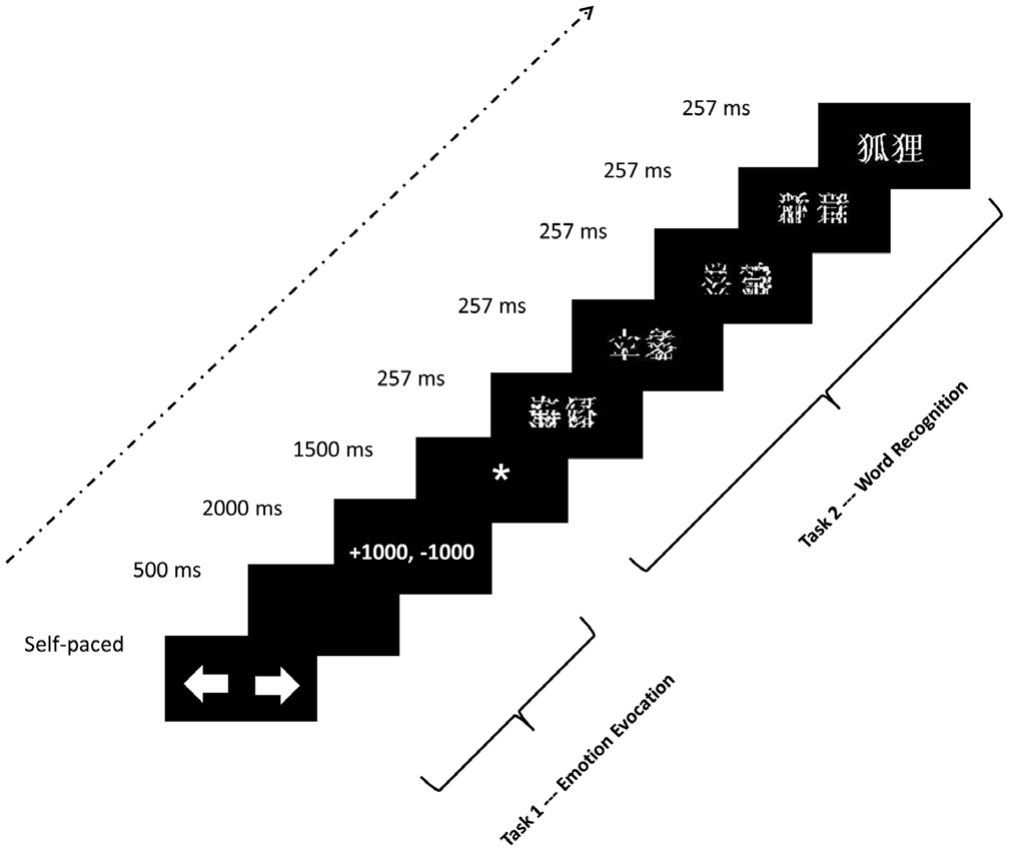
Illustration of the procedure. The emotional induction task and the word recognition task were alternated. In this example, the first 4 stimuli after the fixation in Task 2 are word fragments, and the fifth is a word.

Prior to beginning the experiment, participants were asked to read the experimental instructions carefully. The practice session started after participants indicated that they completely understood the experimental procedure. As illustrated in Figure 1, the experiment included the emotional induction task and word recognition task.

#### 2.4.1. Emotional induction task

This task was used to induce 5 distinct emotional states in participants. Participants were told to compete with another participant, and that they would be paid 50–100 yuan (about 7–15 USDs) if they outperformed their competitor.

At the beginning of this task, a left arrow and a right arrow were displayed on the screen. Participants were instructed to choose one of the arrows, which resulted in either the gain or loss of points. Subsequent to their choice, the results of the competition were displayed for 2000 ms; the participant's score appeared on the left and the pseudo-competitor's score appeared on the right. In the first part of the experiment, we aimed to induce a neutral emotional state (prior to the induction of positive or negative states so as to avoid possible contamination of the neutral state). Each participant was informed in advance that they would be unable to view their point totals and that the system was recording them automatically. Thus, the points presented on the screen after each trial were “0, 0” (i.e., 0 points for the participant and 0 for the pseudo-competitor), where a score of 0 did not mean 0 points were awarded. This was intended to introduce uncertainty as to their real scores, and may further have prevented participants from counting the total points they received. As such, we may exclude the effect of the total points and attribute the evoked emotional states to the points participants received on each trial. In the second part, the points given to the participant and pseudo-competitor were presented on the screen and were expected to induce 4 different degrees of emotional states: 1) +1000,−1000 (highly positive emotion): a participant received 1000 points while the pseudo-competitor lost 1000 points, which was expected to induce highly positive emotion; 2) +1000,+1000 (slightly positive emotion): both the participant and the pseudo-competitor won a 1000-points, which was expected to induce slightly positive emotion, as the participants increased their point total without expanding the difference between their score and that of the pseudo-competitor; 3) −1000,+1000 (highly negative emotion): The participant lost 1000 points while the pseudo-competitor won 1000 points in this condition, which was expected to induce highly negative emotion; 4) −1000,−1000 (slightly negative emotion): Both the participant and pseudo-competitor lost 1000 points in this condition.

The emotional induction task was conducted 36 times for each of the five conditions (180 times in total). The same results were never displayed more than 4 times in a row.

#### 2.4.2. Word recognition task

Stimuli were presented using the rapid stream stimulation procedure [17]–[18] with a stimulus onset asynchrony (SOA) of 250 ms and an inter stimulus interval (ISI) of 0 ms. There was one session of word recognition after each emotional induction; thus, there were 180 word recognition sessions in total. Every session began with the fixation cue “*”, which was presented for 1500 ms to signal participants to press a button as fast and accurately as possible every time when they detected an animal word. Participants were allowed to blink at this cue, but were instructed to suppress blinking during word presentation. Each session consisted of 3 trials with 21 stimuli per session. A trial started with 4 to 6 word fragments (background stimuli), followed by either an animal or non-animal word. Participants were instructed to judge whether the presented word referred to an animal. Every session contained 0∼2 target stimuli (animal words). There were 4∼6 background stimuli presented before and at least 3 background stimuli presented immediately after the non-animal words.

Every non-animal word was repeated 5 times so that the same words were used for every type of emotional state. The numbers of trials for each experimental condition was equal, and presented in pseudorandom order.

Participants alternatively performed the emotional induction task and the word recognition task. They were given four short breaks during the experiment. After the entire experiment, the participants were asked to rate the degree of their emotional state when they saw their results. This rating was completed on a scale spanning from −5 to +5, with negative numbers indicating more negative emotion and positive numbers indicating more positive emotion. Absolute numerical values indicated the intensity of the emotional states. For example, −5 stands for very disappointed, 0 for neutral, +5 for very happy. At debriefing, all participants indicated that they believed they had been competing with another person during the emotional induction task.

### 2.5. Electrophysiological data recording and analysis

Electroencephalographic (EEG) data were recorded using the Scan 4.3 package (NeuroScan, Inc.). Brain electrical activity was recorded from 64 scalp sites using Ag/Agcl electrodes with the left mastoid as the reference. The data were algebraically re-referenced off-line according to the common averaged reference method, which has been proved to be the best procedure to obtain the RP [20]. Bipolar horizontal and vertical electrooculogram (EOG) were recorded for artifact rejection purposes. Electrode impedances were kept below 5 kΩ. The EEG signals were recorded continuously with a band-pass from 0.05 to 100 Hz with a sampling rate of 500 Hz. ERPs were digitally filtered at a low-pass of 30 Hz (24 dB setting).

The EEG data were segmented into 1000 ms epochs, including a 100 ms baseline before the onset of non-animal words under each emotional state. Epochs with artifacts exceeding ±65 μv were automatically rejected.

The RP peak latencies were measured during a 160∼417 ms interval following stimulus onset. A commonly used way to obtain the RP is to subtract ERPs elicited by background stimuli from those elicited by words (which reduces the presence of driving rhythms generated by rapid stream stimulation [18], [26]. However, according to previous studies [19], [29], the RP can be observed clearly by using the rapid stream stimulation paradigm, and so the present study did not use the subtraction method. After determining the mean latency under every emotional state, a time-window centered around the mean latency was considered for measuring mean amplitudes. According to previous studies on the RP [18], [20], [21], [25], this time window ranged ±28 ms around the mean latency under each emotional state.

Repeated measure ANOVAs with the aim of comparing the activity evoked by non-animal words under all 5 kinds of emotion states were performed on a selected sample of 10 electrodes (P1/P2, P5/P6, PO3/PO4, PO7/PO8, O1/O2), which showed a clear RP. These ANOVAs included three factors: emotional state (5 levels: neutral, highly positive, slightly positive, highly negative, slightly negative), hemisphere (2 levels: left, right), and electrode position (5 levels). The Greenhouse-Geisser correction was applied when appropriate. According to previous literature on the RP [18], [20], [21], [25], the PO7 and PO8 electrodes, which showed the strongest RP over each hemisphere, were then chosen for the preset contrasts.

## Results

### 3.1. Behavioral results

Participants' self-rating scores of their emotional states are illustrated in Table 1. In order to directly compare the intensity of the 5 emotional conditions, repeated measure ANOVAs were performed on the absolute values of these rating scores. A significant main effect was shown, *F* (1, 18)  = 19.29,*p*<0.001, indicating a significant difference between different emotional states. Further post hoc analyses showed no significant differences between the rating scores under highly positive and highly negative conditions, and between slightly positive and slightly negative conditions, while there are significant differences between other pairs of comparisons (*ps* <0.001, Bonferroni-corrected). Results suggested that different degrees of emotional states were induced successfully, and that the intensities of the positive and corresponding negative (highly positive vs. highly negative, slightly positive vs. slightly negative) emotional states were well matched.

**Table 1:** Participants' self-rating scores for their emotional states under five conditions (SDs in parentheses).

Points	+1000, −1000	+1000, +1000	0, 0	−1000, −1000	−1000, +1000
Emotional State	Highly Positive	Slightly Positive	Neutral	Slightly Negative	Highly Negative
Ratings	4.29 (0.00)	2.08 (0.71)	0.05 (0.00)	−2.10 (0.71)	−3.87 (0.00)

Performance on the animal words in the word recognition task showed that participants were able to perform the task successfully and intensively, with 98.3% correct responses under the neutral emotional state, 98.1% under the highly positive emotional state, 98.4% under the slightly positive emotional state, 97.9% under the highly negative emotional state, and 98.6% under the slightly negative emotional state.

### 3.2. Electrophysiological data

**Figure 2:**
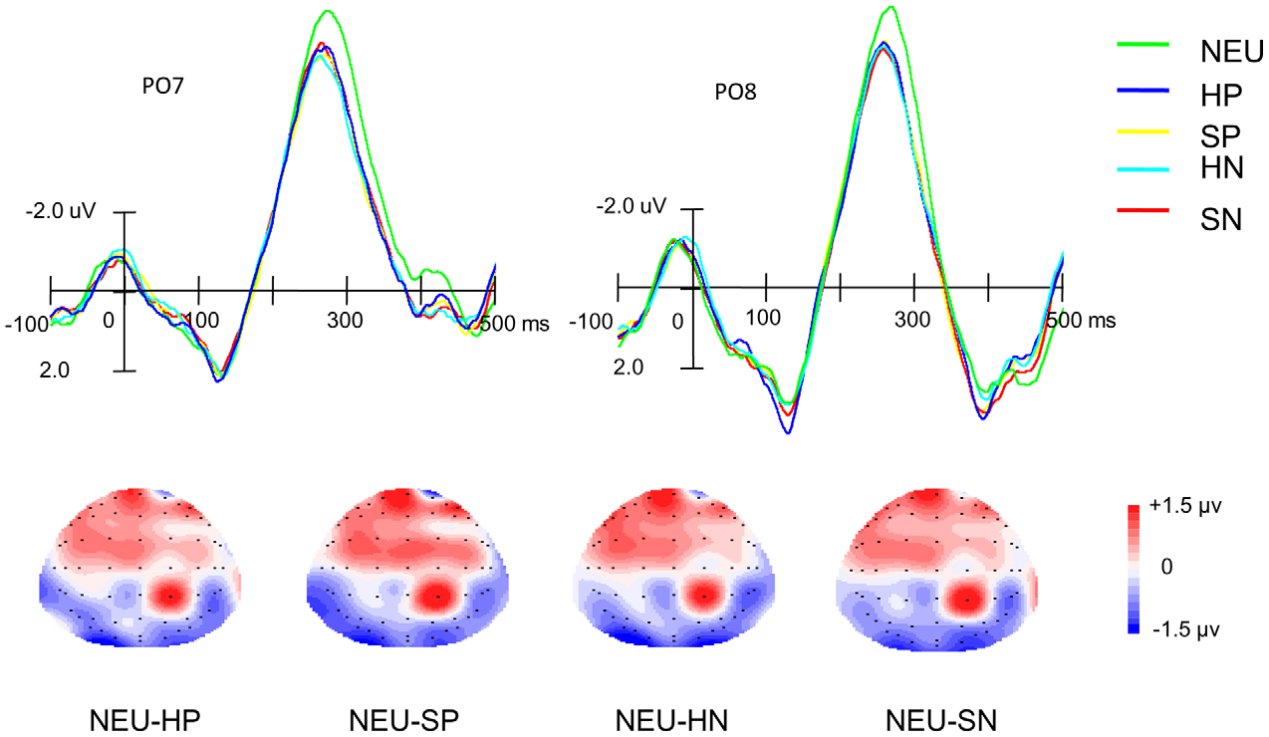
The top panel shows ERPs elicited by word processing under five emotional states at PO7 and PO8 electrodes. The bottom panel shows topographic maps for difference waves between neutral and each of the other four emotional states during the time window of 250–280 ms after stimulus onset. A clear recognition potential (RP) over the occipital scalp can be indentified under the neutral (NEU), highly positive (HP), slightly positive (SP), highly negative (HN) and slightly negative (SN) emotional states, which is highest in the case of the neutral emotional state.

**Figure 3:**
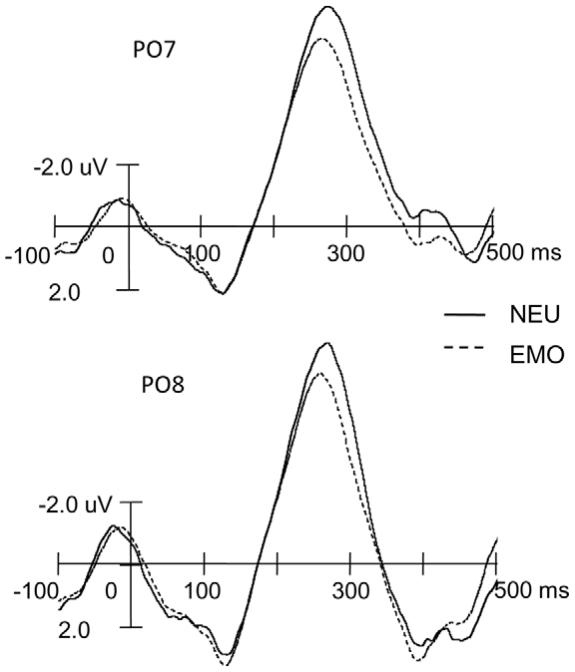
The comparison between neutral (NEU) and other emotional states (EMO).

**Figure 4:**
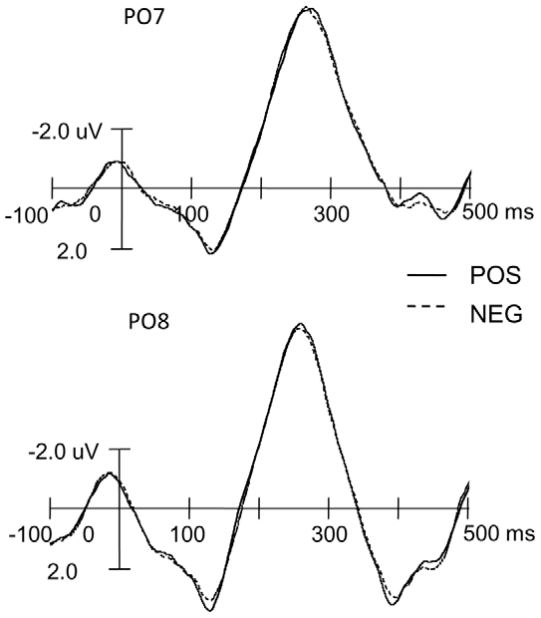
The comparison between positive (POS) and negative (NEG) emotional states.

Figures 2, 3, and 4 display the ERP wave forms evoked by non-animal words under the five emotional states and topographic maps between the neutral and each emotional state. A clear RP, peaking around 260 ms, is observed over the posterior scalp. Visual inspection suggests that its amplitude is larger under the neutral emotional state. Consistent with previous studies on RP [20], [21], [26], [27], the RP showed larger amplitudes over the PO7 and PO8 electrodes. Table 2 summarizes mean amplitudes and peak latencies of RP under all types of emotional states over these two electrodes.

**Table 2:** Mean amplitudes and peak latencies for the recognition potential (RP) evoked by the word processing under five types of emotional states at electrodes that showed the largest RP responses (Standard Deviations were illustrated in parentheses).

	PO7		PO8	
	Amplitude (μV)	Latency (ms)	Amplitude (μV)	Latency (ms)
Neutral	−6.64 (2.56)	276 (20.56)	−6.35 (2.46)	264 (13.45)
Highly positive	−5.67 (2.45)	266 (19.57)	−5.68 (2.52)	257 (12.92)
Slightly positive	−5.59 (2.47)	269 (21.92)	−5.55 (2.19)	257 (16.84)
Highly negative	−5.35 (2.42)	261 (20.24)	−5.53 (2.16)	256 (12.66)
Slightly negative	−5.74 (2.60)	268 (16.76)	−5.42 (2.26)	255 (18.90)

According to a three-way repeated measure ANOVA performed on the mean amplitudes, there was a significant main effect of emotional state [*F* (4, 72)  = 11.25, *p*<0.001], as well as electrode position [*F* (4, 72)  = 43.73, *p*<0.001], but not for hemisphere [*F* (1, 18)  = 1.98, *p*>0.1]. The interaction between emotional states and electrode position was also significant [*F* (16, 288)  =  1.76, *p*<0.05.

As for the peak latencies, results revealed a significant main effect of hemisphere [*F* (1, 18)  = 12.87, *p*<0.001]; that is, the latency of the RP over the right hemisphere (262 ms) was shorter than that over the left hemisphere (276 ms). A significant main effect of electrode position was also observed [*F* (4, 72)  = 6.83, *p*<0.001]. The interaction between emotional state and electrode position was significant [*F* (16, 288)  = 3.69, *p*<0.001], as well as the interaction between hemisphere and electrode position [*F* (4, 72)  = 2.56, *p*<0.05]. However, there was no significant main effect of emotional state [*F* (4, 72)<1].

Consistent with previous studies [20], [21], [26], [27], the largest RP in the present study was observed over the PO7 and PO8. Preset orthogonal contrasts were further performed to reveal differences between RPs under the neutral and other emotional states, the positive and negative emotional states, the highly and slightly positive emotional states, and the highly and slightly negative emotional states over these two electrodes.

For the mean amplitudes of the RP over the PO7 electrode, the orthogonal contrasts revealed significant differences between the neutral and other emotional states [*F* (1, 18)  = 37.76, *p*<0.001], as well as the highly and slightly negative emotional states [*F* (1, 18)  = 5.51; *p*<0.05], but no significant differences between the positive and negative emotional states or the highly positive and slightly positive emotional states [*Fs* (1, 18)<1]. For the mean amplitudes of the RP over the PO8 electrode, the orthogonal contrasts showed no significant differences between different emotional states [*Fs* (1, 18)<1], except for the difference between neutral and other emotional states [*F* (1, 18)  = 9.83, *p*<0.01].

For the peak latencies, similar results were obtained. At the PO7 electrode, the orthogonal contrast revealed a significant difference between the peak latencies of the RP elicited under the neutral and other emotional states [*F* (1, 18)  = 10.89, *p*<0.01] as well as between highly and slightly negative emotional states [*F* (1, 18)  = 4.99, *p*<0.05], but no significant differences between the peak latencies of the RP under positive and negative emotional states and between highly positive and slightly positive emotional states [*F* (1,18)  = 2.93, *p*>0.1; *F* (1,18)  = 1.00, *p*>0.1, respectively]. At the PO8 electrode, the orthogonal contrast showed no significant difference between different conditions [*Fs* (1, 18)<1], with the exception of the difference between the peak latencies of the RP under neutral and other emotional states [*F* (1, 18)  = 13.60, *p*<0.01].

## Discussion

To investigate if and how word processing is modulated by emotional states, the present study first induced emotional states by making participants compete with a pseudo-participant, and then required them to complete a word recognition task. The results demonstrated that the RP, a good index of early word processing, is sensitive to an individual's emotional states. The results are discussed below.

### 4.1. Is word processing modulated by emotional states?

In the present experiment, a clear-cut RP was found in the word recognition task. This further suggests that the RP can be elicited by recognizable stimuli. Consistent with the findings of Hinojosa et al. (2001) [25], which showed that the RP elicited by content words had a similar topographical distribution over two hemispheres, the present study also found no difference between the mean amplitudes of the RP over two hemispheres. This is likely due to the fact that nouns, a sub-category of content words, were used in the present study. Furthermore, compared to the left hemisphere, the peak latency of RP was shorter over the right hemisphere. As revealed by previous studies [20]–[21], [26]–[27], the RP has a larger topographical distribution over the occipital scalp and originates from the lingual gyrus and fusiform gyrus, which play an important role in word recognition. Accordingly, the present findings indicate that the early stage of processing Chinese nouns may first recruit the right lingual gyri and fusiform gyri for spatial structure analysis of Chinese words, and then the contralateral brain areas for higher levels of word processing.

More interestingly, compared with the neutral emotional state, word recognition under the positive and negative emotional states elicited a smaller RP over each hemisphere. The difference mainly distributed over the occipital scalp. A reduced RP reflects less cognitive resource is required to process concrete words relative to abstract words [21]. The present results suggest that less cognitive resources may be allocated for participants to perform a cognitive task such as word recognition under positive and negative emotional states [6], [28]. Another possibility is that the emotional induction task requires more attentional resources, which leads to fewer resources for subsequent word processing and thus a smaller RP. However, the present study can not differentiate which possibility is more reasonable.

In addition, a significant effect of emotion was observed on the RP peak latency. Specifically, a shorter peak latency of the RP was found under emotional states. The reduced latency reflects a faster word processing [18]–[21], [29]. The finding of shorter peak latency of the RP is also consistent with previous studies demonstrating that words with different emotional valences were processed faster than neutral words when participants performed a lexical decision task [2, 30–32). This may indicate that the early stage of word processing is faster under negative or positive emotional states. However, participants were not required to respond to the words of interest in the present study, so it remains an open question whether the finding of shorter peak latency of the RP can be corroborated by the reaction time measure.

### 4.2. Do different degrees of emotional states have the same effect on RP?

In our daily experience, cognitive processing is relatively more efficient under a peaceful emotional state than under an intense emotional state. Indeed, emotional background with high arousal was found to impede word processing but that with low arousal was not [12]. Therefore, another interesting question is whether different degrees of emotional states have different effects on the RP.

The results of the present study showed that, over the left hemisphere, significant differences were observed in the mean amplitude and peak latency of the RP elicited under different degrees of negative emotional states. Specifically, a smaller RP with earlier peak latency was elicited under the highly negative emotional state over the left hemisphere than that under the low negative emotional state. In other words, under the highly negative emotional state, word processing was faster and required fewer attentional resource over the left hemisphere. No such differences were found over the right hemisphere. As mentioned earlier, the right lingual gyrus and fusiform gyrus may be recruited earlier for sub-word level processing, followed by the left lingual gyrus and fusiform gyrus for word level processing. This finding of an asymmetry between the two hemispheres under different degrees of negative emotional states suggests that the early stage of word level processing is more sensitive to negative emotional states.

In terms of positive emotional states, no difference was found in either the mean amplitude or peak latency of the RP under different degrees of emotional intensity. That is, positive emotional states of different intensity had similar effects on word recognition. However, it is curious that over the left hemisphere, different degrees of negative emotion had different effects on word processing, while positive emotions did not. There are two possible explanations for this effect. First, even though in general there may be no difference between the effect of the positive and negative emotions on word processing, the early stage of word processing may be more sensitive to negative emotions. Some previous studies also showed that negative emotional information [2], [5], [9], [12], [33] has a stronger effect on word processing than positive information. Second, though highly positive and highly negative emotions were well matched in the present study, previous research [23] suggests that losing money may induce stronger emotional experiences than winning the same amount of money. In other words, the disparity between highly negative and slightly negative emotions may be larger than that between highly positive and slightly positive emotions in our experiment. In the present study, that disparity may have led to the different effects on word processing under negative emotions of different intensity. Therefore, it may be possible that when the disparity in intensity between two types of positive emotional states increases, significant effects of different degrees of positive emotion may be observed on the RP. Further studies may examine this possibility.

Furthermore, no difference was observed between RPs under positive and negative emotional states, which indicates that positive and negative emotions have similar effects on word processing. This is inconsistent with the findings in several previous studies. Some of these studies found that negative emotion has a greater effect on word processing than positive emotion [2], [5], [9], [34], while others drew the opposite conclusion [35]–[36]. The inconsistency between the present study and previous research may be partly attributed to the different degrees of emotional arousal between positive and negative emotions. In previous studies, emotional states were mostly rated by participants themselves with strong subjectivity; however, in the present study, 5 degrees of emotion were induced in a pseudo-competition which immediately preceded the word-recognition task. This method of inducing emotional states may have resulted in a better correlation between the emotional states and performance on the word-recognition task.

To summarize, the influence of emotional states on word processing manifests as a smaller RP with an earlier peak latency. In addition, positive and negative emotional states have similar effects on word processing, but positive and negative emotions of differing degree have different effects. The effects of positive emotions of varying degree on word processing are similar, but compared to low negative emotion, highly negative emotion facilitates word processing over the left hemisphere.
